# Threshold Effect of Time to Admission on Long-Term Mortality in Geriatric Hip Fractures: A 24-H Critical Window Identified

**DOI:** 10.3390/jcm15020752

**Published:** 2026-01-16

**Authors:** Bin-Fei Zhang, Ming-Xu Wang

**Affiliations:** 1 School of Public Health, Xi’an Jiaotong University, No. 76 Yanta West Road, Xi’an 710061, China; beephoe@stu.xjtu.edu.cn; 2 Department of Joint Surgery, Honghui Hospital, Xi’an Jiaotong University, No. 555 Youyi East Road, Xi’an 710054, China

**Keywords:** hip fracture, elderly, delayed, time to admission, risk, mortality, prognosis

## Abstract

**Objective:** This study aimed to investigate the association between time to admission (TTA) and long-term mortality in patients with hip fractures, enabling surgeons to assess individual risks and prevent adverse outcomes. **Methods:** Demographic and clinical data of patients with hip fractures were obtained from medical records in our hospital. Patients aged 65 years or older were included. TTA was defined as the time from injury to first presentation at our institution. The primary outcome was long-term all-cause mortality. The regular multivariate Cox regression, restricted cubic spline, and two-piecewise model were used to explain the linear and curvilinear association between TTA and long-term mortality. The analyses were performed using EmpowerStats and R. **Results:** A total of 2361 patients were included in our study. There were 743 males and 1618 females, with a mean age of 79.44 ± 6.71 years. There were 1745 intertrochanteric fractures and 616 femoral neck fractures. We divided the patients into four groups according to TTA distribution: TTA ≤ 6 h, 6 h < TTA ≤ 12 h, 12 h < TTA ≤ 24 h, and TTA > 24 h, and the corresponding long-term mortality rates were 254 (25.53%), 85 (32.20%), 127 (32.56%), and 267 (37.50%). A curvilinear association was observed between TTA delay and long-term mortality in geriatric hip fractures, with 24 h serving as an inflection point. When TTA was less than 24 h, every one-hour increase in TTA was associated with a 1.6% increase in long-term mortality (HR = 1.016, 95% CI: 1.008–1.024; *p* < 0.001). When TTA exceeded 24 h, the long-term mortality risk showed no significant further increase with TTA (HR = 1.000, 95% CI: 1.000–1.000; *p* = 0.531). **Conclusions:** This study suggests that delayed admission is associated with a worse prognosis, and the mortality risk increases by approximately 1.6% per hour of delay within the first 24 h, after which the risk appears to stabilize. The first 24 h post-injury may represent a critical window for intervention.

## 1. Introduction

Hip fractures are common osteoporotic fractures, and early surgery is advised for patients [1]. Guidelines recommend a corresponding surgery time of 36 or 48 h after admission [2,3].

The surgical delay is a risk factor for postoperative patients’ death and complications. Furthermore, delayed diagnosis and admission for hip fractures are associated with an increased risk of specific pre-mortem complications. These include systemic complications such as deep vein thrombosis, pulmonary infections, and pressure sores. These complications can initiate a cascade of decline, ultimately contributing to the elevated long-term mortality observed in this patient population [4]. However, due to the rich medical resources in developed countries, delays in hospitalization are rare after injury, so most observational studies consider the association of the time from admission to surgery with prognosis alone, while ignoring the delay time to admission [5,6], called TTA [7]. In a retrospective study from He et al., they found that the proportions of patients hospitalized on the day of injury, within the first 1 day, and within the first 2 days were 25.4%, 54.7%, and 66.3%, respectively, among which 12.6% of patients received medical treatment at the hospital more than one week after the injury [8]. Therefore, the TTA delay was not rare.

Due to a lack of relevant medical knowledge or insufficient social support, some patients experience long delays before hospitalization. Several previous studies have investigated the relationship between TTA delay and the overall prognosis of elderly patients with hip fractures. It was reported that TTA delay caused by transfer from the first consultation was associated with an increase in perioperative complications and mortality rate [9], and it was a risk factor for the increase in 30-day postoperative mortality rate [10]. At the same time, TTA delay was associated with a 9% decrease in the post-discharge survival rate and a 7% decrease in the 1-year postoperative survival rate [11], and a TTA delay of more than one week was significantly associated with a 76% increase in the 1-year mortality rate [8]. In our previous study, we found that each 1 h increase in TTA was associated with a 9% increase in the incidence of 1-year mortality when TTA < 9 h [7].

While in-hospital care quality predominantly influences short-term outcomes, we hypothesize that the pre-hospital phase, indexed by the TTA, serves as a critical window that reveals a patient’s underlying vulnerability. We propose that TTA is a composite marker of socio-medical frailty, and that its association with mortality extends into the long term, reflecting the enduring impact of these baseline deficits. However, short-term results on the relationship between TTA and postoperative mortality rate are insufficient to provide strong evidence for clinical practice. Therefore, the primary purpose of this study is to investigate the association between TTA time and long-term mortality, which may help surgeons assess individual risks and inform strategies to mitigate adverse outcomes.

## 2. Materials and Methods

### 2.1. Study Design

In this retrospective cohort study, we included older adults with hip fractures identified in medical records at Honghui Hospital of Xi’an Jiaotong University from 1 January 2015, to 30 September 2019. The Ethics Committee of Honghui Hospital approved this retrospective study (Xi’an Jiaotong University, No. 202201009), and the Institutional Review Boards of Honghui Hospital waived the requirement for informed consent from participants. The study has been reported in accordance with the STROCSS 2021 guidelines [12].

### 2.2. Participants

Demographic and clinical data of the patients were obtained from their original medical records. The inclusion criteria were as follows: (1) Age ≥ 65 years; (2) X-ray or computed tomography (CT) diagnosis of the femoral neck or intertrochanteric fracture; (3) Patients who received surgical treatment in hospitalization; (4) Availability of in-hospital clinical data; (5) Patients or their families could be contacted by telephone.

The exclusion criteria were as follows: (1) the presence of complex injury, such as concurrent fractures of other major bones, soft tissue injuries and visceral injuries; (2) severe multiple injuries caused by a car accident or falling from a height, with the patient in a critical condition upon admission; (3) the specific time of injury to admission and operation is not recorded in the medical records or cannot be calculated; (4) the phone number on the front page of the patient’s medical record is incorrect, and the patient cannot be reached, or the patient’s family members have changed the phone number, or the wrong phone number is dialed and the person contacted is neither the patient nor a family member; (5) patient survival status at the follow-up time was unavailable for reasons unrelated to the study or hip fracture outcomes; (6) the patient’s death was due to non-natural causes (e.g., accident, homicide, suicide, or other unintended external events).

### 2.3. Hospital Treatment

After the patient was admitted to the department from the emergency room or outpatient clinic, surgical treatment was scheduled once no contraindications were detected during the patient’s preoperative evaluation. The treatment of hip fractures is divided into three types, based on the fracture type and AO/OTA classification [13], patient age, and activity level: closed reduction and internal fixation (CRIF/ORIF), hemiarthroplasty (HA), and total hip arthroplasty (THA). For patients with intertrochanteric fractures of the femur, CRIF/ORIF was typically adopted [14]. For patients with femoral neck fractures, those with a high activity level were usually offered THA. If the patient was elderly and had a low activity level, HA treatment was selected [15]. Senior surgeons perform all surgical procedures.

### 2.4. Variables

The variables collected in this study were as follows: age, sex, injury mechanism, fracture classification, hypertension, diabetes, coronary artery disease (CHD), arrhythmia, hemorrhagic stroke, dementia, ischemic stroke, cancer, associated injuries, chronic obstructive pulmonary disease (COPD), hepatitis, gastritis, treatment strategy, TTA, time to operation, operation time, blood loss, stay in hospital, follow-up, mortality. All variables listed and defined in this section were collected as candidate predictors and were subjected to univariate analysis to assess their potential association with the primary outcome of long-term mortality.

The endpoint event was long-term mortality after operation duration, and the independent variable was TTA. TTA was rigorously defined as the time elapsed (in hours) from the documented time of injury to the time of the patient’s first registration at our institution (Honghui Hospital). Registration time was recorded in the emergency department or the outpatient clinic, whichever was the point of first contact. All time data were extracted from the patients’ original medical records, which were completed in real time by medical staff in the emergency department, outpatient clinic, and inpatient wards. This definition encompasses the total pre-hospital delay, including any time spent at other referring hospitals for transferred patients. To ensure data quality, cases with missing, ambiguous, or contradictory time records for injury or admission were strictly excluded, according to exclusion criteria 3).

### 2.5. Follow-Up

Patient follow-up was conducted by telephone from March 2022 to April 2022. This resulted in a variable follow-up duration ranging from approximately 2.5 years (for patients enrolled in September 2019) to 7 years (for patients enrolled in January 2015).

### 2.6. Statistics Analysis

Continuous variables are reported as mean ± standard deviation (SD) or median (with minimum and maximum values), and categorical variables are presented as frequencies and percentages. We used the χ^2^ test, the One-way ANOVA test, or the Kruskal–Wallis H test to assess differences among the different TTA subgroups. A combination of clinical relevance and statistical criteria guided the selection of covariates for adjustment in the multivariate Cox regression model. Firstly, we included variables identified as important prognostic factors in the existing literature on hip fracture mortality [16,17]. Secondly, we considered variables that showed an association with long-term mortality in our univariate analysis (*p* < 0.1). To mitigate multicollinearity among the candidate adjustment variables, we assessed the Variance Inflation Factor (VIF). Variables with VIFs greater than 4 were considered to indicate severe multicollinearity and were carefully evaluated; highly correlated variables were removed to preserve model stability and parsimony. The final adjusted model contained only variables with VIF values below this threshold. This strategy ensured that the model was adjusted for well-known prognostic factors while also exploring the role of other variables within our specific dataset.

We used a multivariate Cox regression model to test the association between TTA and long-term mortality. To test the robustness of our results, we converted TTA into a categorical variable according to the subgroups. We calculated the *P* for trend to verify the TTA results as a continuous variable and to examine the possibility of nonlinearity. To account for the nonlinear relationship between TTA and long-term mortality, we used a Cox regression with a restricted cubic spline and smooth curve fitting. Additionally, a two-piecewise Cox regression model was employed to identify the inflection point and further explain the nonlinearity. To assess the stability and potential overfitting of the inflection point identified by the two-piecewise Cox regression model, we performed an internal validation using bootstrap resampling with 1000 replicates. The 95% confidence interval (CI) for the inflection point was derived from the bootstrap distribution’s percentiles.

Additionally, we present Kaplan–Meier survival curves to illustrate trends across TTA subgroups. A sensitivity analysis was performed by restricting the outcome to mortality events occurring within the first 2 years post-injury to assess the impact of variable follow-up time.

All analyses were performed using the stats package in R 4.5.0 (http://www.R-project.org, R Foundation) and EmpowerStats 4.2 (http://www.empowerstats.com, X&Y Solutions Inc., Boston, MA, USA). Hazard ratios (HR), odds ratio (OR) and 95% CI were calculated. A *p*-value < 0.05 (two-sided) was considered to represent statistical significance.

## 3. Results

### 3.1. Patient Characteristics

Of 2839 patients screened, 2361 were included in the final analysis. A total of 478 patients were excluded due to loss to follow-up (primarily because of incorrect telephone numbers or unavailability, aligning with exclusion criteria #4 and #5), resulting in a successful follow-up rate of 83.2%. To assess potential selection bias, we compared the baseline characteristics of the 2361 included patients with those of the 478 patients lost to follow-up. As shown in Supplementary Materials Table S1, there were no statistically significant differences between the two groups except for the stay in hospital.

The general information and baseline characteristics of patients are shown in Table 1. There were 743 males and 1618 females, with a mean age of 79.44 ± 6.71 years. There were 1745 intertrochanteric fractures and 616 femoral neck fractures. We divided the patients into four groups based on TTA distribution: TTA ≤ 6 h, 6 h < TTA ≤ 12 h, 12 h < TTA ≤ 24 h, and TTA > 24 h. The number of patients was 995, 264, 390, and 712 in each group, respectively, and the corresponding long-term mortality rates were 254 (25.53%), 85 (32.20%), 127 (32.56%), and 267 (37.50%).

### 3.2. Univariate Analysis of the Association Between Variables and Long-Term Mortality

Based on the univariate analysis of the criteria of *p* < 0.1 and the previously reported factors and the result of VIF, we found these confounding factors to adjust: age, sex, CHD, arrhythmia, ischemic stroke, cancer, dementia, COPD, hepatitis, and time to operation, fracture classification, and treatment strategy (Supplementary Materials Table S2).

### 3.3. Multivariate Analysis Between Preoperative TTA and Long-Term Mortality

We used a Cox regression model to assess the association between TTA and long-term mortality. In Table 2, TTA was associated with long-term mortality (HR = 1.000; 95% CI: 1.000–1.000; *p* = 0.040) in the adjusted model II. When we divided TTA into different subgroups, the results were unstable. In the fully adjusted model, compared to the TTA ≤ 6 h subgroup, the 6 h < TTA ≤ 12 h subgroup was not associated with long-term mortality (HR = 1.223; 95% CI: 0.955–1.568; *p* = 0.111), the 12 h < TTA ≤ 24 h subgroup was associated with long-term mortality (HR = 1.250; 95% CI: 1.008–1.550; *p* = 0.042), the TTA > 24 h subgroup was associated with long-term mortality (HR = 1.489; 95% CI: 1.249–1.776; *p* < 0.001).

### 3.4. Curve Fitting and Analysis of the Threshold or Saturation Effect

**Figure 1:**
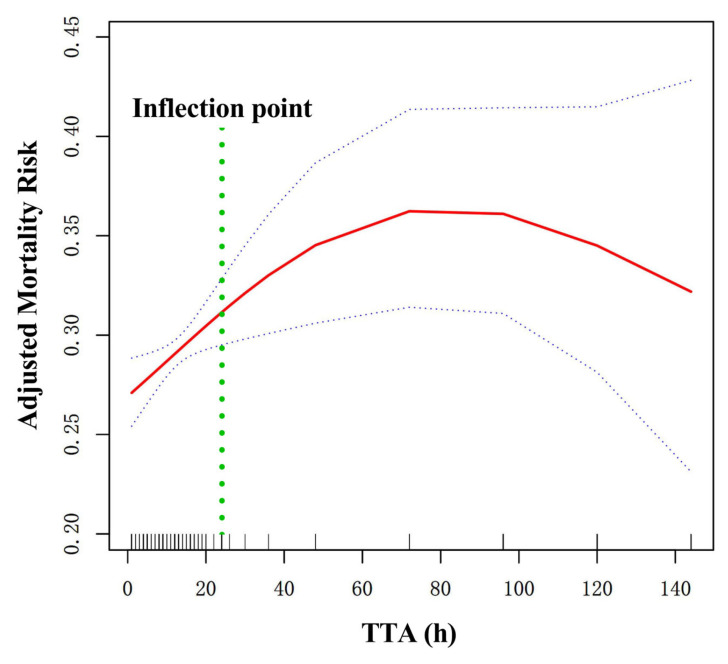
Curve fitting between TTA and long-term mortality. They were adjusted for age, sex, CHD, arrhythmia, ischemic stroke, cancer, dementia, COPD, hepatitis, time to operation, stay in hospital, fracture classification, and treatment strategy. The red solid line means the fitting line, and the blue dashed line means the 95% CI. The green dashed line means the inflection point.

As shown in Figure 1, a curvilinear correlation was observed between TTA and long-term mortality after adjusting for confounding factors. The two-piecewise Cox regression model indicated that the association pattern changed at approximately 24 h, as shown in Table 3. The bootstrap internal validation based on 1000 replicates supported the stability of this inflection point, yielding a 95% CI of 21–28 h.

For TTA < 24 h, there was a correlation between TTA and long-term mortality. For every 1 h increase in TTA, long-term mortality increased by 1.6% (HR = 1.016, 95% CI: 1.008–1.024; *p* < 0.001). For TTA ≥ 24 h, the risk of long-term mortality reached a plateau, showing no significant further increase with TTA (HR = 1.000, 95% CI: 1.000–1.000; *p* = 0.531).

### 3.5. The Kaplan–Meier Survival Curves

**Figure 2:**
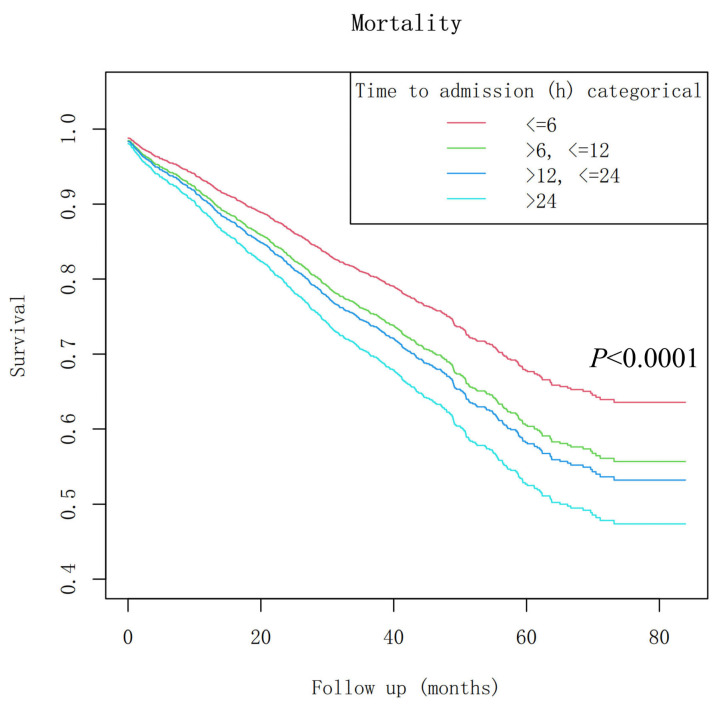
The Kaplan–Meier survival curve by TTA subgroups, with the identified inflection point at 24 h.

The Kaplan–Meier survival curves for the TTA subgroups, with the inflection point at 24 h, are shown in Figure 2.

### 3.6. Stratification Analysis

Stratification analyses were conducted to explore whether the association between TTA and mortality was consistent across key patient subgroups, as shown in Table 4. While the overall nonlinear relationship was frequently observed, the specific inflection points varied widely, with estimates ranging from 2 to over 300 h. This considerable variation is likely attributable to the substantially reduced statistical power within these smaller subgroups, making precise threshold estimation unreliable. Therefore, the primary analysis based on the entire cohort, which identified a 24 h threshold, should be considered the most robust finding.

### 3.7. Sensitivity Analysis

A sensitivity analysis confined to 2-year mortality revealed a consistent and significant nonlinear association with TTA, confirming that the risk associated with delayed admission is evident in both the short- and long-term. As shown in Supplementary Materials Table S3, the inflection point is 17 h (95% CI: 9–22 h). For TTA < 17 h, every one-hour increase in TTA was associated with a 3.2% increase in mortality of patients (OR = 1.032, 95% CI: 1.013–1.051; *p* = 0.001). For TTA > 17 h, the risk of mortality reached a plateau, showing no significant further increase with TTA (OR = 1.000, 95% CI: 1.000–1.001; *p* = 0.487).

## 4. Discussion

This study found that delayed admission was associated with a worse prognosis and a curvilinear relationship between TTA delay and long-term mortality in geriatric hip fractures. The 24 h TTA was an inflection point in the curve fitting. Each one-hour increase in TTA was associated with a 1.6% increase in long-term mortality when TTA < 24 h. Long-term mortality peaked when TTA > 24 h, and mortality risk showed no significant further increase with TTA.

Several studies investigated the association between TTA and the prognosis of elderly patients with hip fractures. A prospective cohort study involving 78 patients demonstrated a TTA delay of 3.45 days, with each one-day delay in TTA associated with a 22% increase in 30-day mortality [10]. This result underscores the need to raise awareness, enhance the referral process, and establish hospital protocol-based care. Additionally, a study involving 343 patients from Brazil found that the mean TTA time was 3 days. TTA delays were associated with a 9% decrease in post-discharge survival and a 7% decrease in 1-year postoperative survival [11].

Our study identified 24 h as a critical threshold for TTA. While identified through data-driven methods, this threshold aligns with established clinical paradigms that emphasize the first 24 h as crucial for resuscitation and initial management in trauma care [18,19,20], especially Advanced Trauma Life Support guidelines [21]. The bootstrap confidence interval around this point was narrow, suggesting it is a stable finding within our population. Although stratified analyses revealed varying point estimates for the threshold, this is an expected consequence of limited subgroup power and should not detract from the primary, clinically coherent finding of a 24 h window. Future studies with larger cohorts are needed to validate whether this threshold holds across all patient subtypes.

The demographic profile of our cohort, with a mean age of 79 years and a predominance of female patients (70%), is characteristic of a geriatric population with hip fractures. Furthermore, the high proportion of extracapsular fractures (74%) aligns with the previously described fracture pattern in Asian populations. This confirms the representativeness of our sample for the target demographic in this region [22,23].

It is crucial to interpret the identified 24 h time point as an exploratory finding from this retrospective dataset. While it provides a clinically valuable reference and aligns with established practice paradigms, it should not be viewed as a rigid, universal threshold. Our subgroup analysis indicated that the inflection point differed substantially by fracture type, with estimates of 48 h for femoral neck fractures and 11 h for intertrochanteric fractures. This suggests that the ‘critical window’ for intervention may be pathology-specific. While this underscores the complexity of the relationship, the smaller sample sizes within each subgroup, particularly for femoral neck fractures, yield less precise estimates (wide confidence intervals). Therefore, the primary 24 h threshold from the complete cohort analysis should be interpreted as a population-average estimate, and future studies are needed to validate and refine fracture-specific time targets.

A clinically significant finding was that a substantial proportion of our cohort (30.1%) experienced delays to admission exceeding 24 h. This highlights a critical area for quality improvement in pre-hospital care and social support networks for geriatric patients with hip fractures in our region. Furthermore, we acknowledge that fracture type (intracapsular vs. extracapsular) could act as a confounder, as it influences both the clinical presentation (potentially affecting TTA) and the baseline mortality risk. However, our multivariate models were rigorously adjusted for fracture type, and the persistent, strong association between TTA and mortality after this adjustment lends greater credibility to a direct effect of the delay itself.

Although TTA is a time-window indicator, this delay time does not simply reflect the issue of medical resource distribution. Instead, TTA is a concrete manifestation of the structural defects in the social support system regarding hip fractures in the elderly. TTA serves as a mirror for examining the integrity of the social support system, reflecting multidimensional social support elements such as family structure, community services, and medical security. After the injury, family support provides the elderly with much-needed care. Despite a significant decline in the proportion of older adults living with their children, the number of older adults living alone is increasing [24]. If a single older adult is injured and their children cannot arrive in time, the proportion of patients sent to the hospital by family members within a short time is very low. Even if the elderly live with their children, if family members have an insufficient educational level and lack awareness of fractures, they will not ask for medical treatment promptly. If the patient lives in the countryside or the suburbs and has limited transportation, family members may be unable to transfer the patient to the hospital shortly after the injury. As the second line of defense, community services serve as a substitute for early medical transportation for elderly patients with hip fractures. Whether the community has volunteers specifically for elderly widows and widowers, whether staff members conduct regular home visits to the elderly population, and whether intelligent fall-monitoring devices are configured for high-risk fall populations are all very important for early detection of older adults who have fallen. Reconstructing the social support network requires a multidimensional intervention: establishing a three-level response mechanism of “family-community-hospital”, training professional elderly emergency responders at the community level, configuring intelligent fall-monitoring devices, and allowing contracted family doctors to directly initiate the referral process in emergencies. Patients with a shorter TTA may have broader social support, enabling them to be transferred to the hospital for treatment in the early stages after injury. At the same time, patients with a shorter TTA can receive more timely standardized anticoagulant and fluid replacement treatment, complete preoperative examinations earlier, and have relatively higher social support after surgery. Therefore, patients with a short TTA have better postoperative recovery.

The principal finding of this study, a strong association between TTA and long-term mortality, can be interpreted by reconceptualizing TTA beyond a simple time metric. We posit that delayed admission is a proxy for a ‘frailty phenotype’ encompassing poor social support, cognitive decline, and limited access to care. This phenotype not only delays presentation but also portends a persistently higher risk of death over the years [25]. Thus, TTA acts as an early, readily available indicator of a patient’s long-term trajectory, capturing risks that traditional in-hospital variables might miss.

Social support is an important protective factor for the prognosis [26,27,28]. Zhang et al. investigated whether preinjury perceived social support moderated the association between pre- and postinjury functional status after hip fracture in older adults [25]. They found that preinjury perceived social support moderates the association between pre- and postinjury functional status in older adults experiencing a hip fracture. Preinjury limitations in activities of daily living were associated with greater postinjury activities among people with and without preinjury perceived social support. Older adults with pre-existing limitations in instrumental activities of daily living, without social support, are at high risk of continued or worsening activity limitations [25].

As our definition of TTA includes time spent during inter-hospital transfers, the delays we observed are likely reflective of broader systemic issues in the pre-hospital pathway. Based on our findings, we propose the following testable hypotheses and potential intervention directions for future research aimed at optimizing TTA: (1) Whether community-based education (e.g., via posters and lectures) to train family members in recognizing the classic signs of hip fracture (limb shortening, external rotation, pain) can reduce pre-hospital delay. (2) Whether optimizing emergency triage to prioritize suspected elderly fracture patients and equipping ambulances with portable X-ray for pre-hospital diagnosis can effectively shorten TTA. (3) Whether incorporating TTA as a key performance indicator into public health surveillance and establishing a regional trauma network can systematically reduce delays.

This study has several key strengths. First, its large sample size provides robust statistical power to detect clinically significant associations. Second, it focuses on long-term mortality, a patient-centered outcome often overlooked in favor of short-term endpoints, while also including a sensitivity analysis of 2-year mortality. Third, the application of advanced statistical modeling—restricted cubic splines and piecewise regression—allowed us to identify and characterize a critical 24 h threshold, thereby capturing a complex nonlinear relationship that moves beyond simple linear assumptions. Furthermore, extensive sensitivity and subgroup analyses were performed to verify the robustness of the results. Most importantly, by conceptualizing TTA as a potential marker of underlying socio-medical frailty, this work shifts the focus to the often-neglected pre-hospital phase and provides a framework for holistic patient assessment.

Our findings must be interpreted considering several limitations. First, the retrospective observational design precludes causal inference and is susceptible to unmeasured confounding. Key prognostic factors such as formal frailty assessments, detailed socioeconomic status, and pre-injury functional capacity were not available. Second, although we defined TTA precisely and excluded cases with unclear records, the exact time of injury might be subject to recall bias, especially in patients without witnesses. Such non-differential misclassification would typically bias the effect estimates toward the null, suggesting that our reported hazard ratio of 1.6% per hour might be a conservative estimate. Third, although the loss to follow-up rate (16.8%) is acceptable and the baseline characteristics were broadly similar between groups, it may still introduce potential bias. Future analyses could consider employing methods such as inverse probability weighting to further assess and adjust for the potential impact of loss to follow-up. A further limitation is that patients lost to follow-up had a slightly longer mean hospital stay, which may introduce a conservative bias if it reflects unmeasured comorbidities, potentially attenuating the observed association between TTA and mortality. Fourth, although we adjusted for known confounders using multivariable models, residual confounding may persist. For instance, we lacked detailed assessments of patient frailty, cognitive status, and socioeconomic status—unmeasured factors that could influence both admission delay and long-term outcomes. Finally, our cohort’s high prevalence of extracapsular fractures may limit the generalizability of the exact 24 h threshold to populations with different fracture type distributions. Future studies should consider employing more advanced methods, such as propensity score matching or instrumental variable analysis, to further control for confounding and more rigorously assess the potential causal relationship between TTA and mortality.

Future research should prioritize prospective studies that collect granular data on frailty, cognitive status, and social support to disentangle whether TTA is a causal risk factor or primarily a marker of underlying vulnerability. The differing inflection points observed for femoral neck and intertrochanteric fractures warrant validation in larger, fracture-specific cohorts. Ultimately, our findings underscore the need to develop and evaluate targeted pre-hospital interventions—such as community-based fall response networks or streamlined referral pathways—aimed at reducing delays within this critical early window to improve long-term outcomes for frail older adults.

## 5. Conclusions

This study identifies the first 24 h post-injury as a critical window in geriatric hip fractures, with each hour of admission delay within this period increasing long-term mortality risk by 1.6%. Time to admission serves as a marker of socio-medical frailty, reflecting gaps in pre-hospital care and social support. These findings highlight the urgent need for community-based strategies to reduce early delays and improve outcomes for this vulnerable population.

## Figures and Tables

**Table 1:** The general information and baseline characteristics of patients.

TTA Subgroups	TTA ≤ 6 h	6 h < TTA ≤ 12 h	12 h < TTA ≤ 24 h	TTA > 24 h	*p*-Value ^†^	*p*-Value *
**No. of patients**	995	264	390	712		
**TTA (h)**	3 (1–6)	9 (7–12)	24 (13–24)	144 (26–5040)	<0.001	<0.001
**Age (y)**	79.10 ± 6.52	78.94 ± 6.59	80.07 ± 7.12	79.76 ± 6.76	0.028	0.033
**Sex**					0.449	-
Male	300 (30.15%)	78 (29.55%)	129 (33.08%)	236 (33.15%)		
Female	695 (69.85%)	186 (70.45%)	261 (66.92%)	476 (66.85%)		
**Injury mechanism**					0.001	-
Falling	961 (96.58%)	250 (94.70%)	384 (98.46%)	691 (97.05%)		
Accident	30 (3.02%)	14 (5.30%)	5 (1.28%)	12 (1.69%)		
Other	4 (0.40%)	0 (0.00%)	1 (0.26%)	9 (1.26%)		
**Fracture classification**					<0.001	-
Intertrochanteric fracture	789 (79.30%)	204 (77.27%)	281 (72.05%)	471 (66.15%)		
Femoral neck fracture	206 (20.70%)	60 (22.73%)	109 (27.95%)	241 (33.85%)		
**Hypertension**					0.02	-
No	520 (52.26%)	135 (51.14%)	219 (56.15%)	333 (46.77%)		
Yes	475 (47.74%)	129 (48.86%)	171 (43.85%)	379 (53.23%)		
**Diabetes**					0.184	-
No	808 (81.21%)	206 (78.03%)	326 (83.59%)	562 (78.93%)		
Yes	187 (18.79%)	58 (21.97%)	64 (16.41%)	150 (21.07%)		
**CHD**					0.965	-
No	481 (48.34%)	125 (47.35%)	192 (49.23%)	348 (48.88%)		
Yes	514 (51.66%)	139 (52.65%)	198 (50.77%)	364 (51.12%)		
**Arrhythmia**					0.025	-
No	702 (70.55%)	177 (67.05%)	271 (69.49%)	454 (63.76%)		
Yes	293 (29.45%)	87 (32.95%)	119 (30.51%)	258 (36.24%)		
**Hemorrhagic stroke**					0.002	-
No	984 (98.89%)	255 (96.59%)	386 (98.97%)	688 (96.63%)		
Yes	11 (1.11%)	9 (3.41%)	4 (1.03%)	24 (3.37%)		
**Ischemic stroke**					<0.001	-
No	757 (76.08%)	194 (73.48%)	287 (73.59%)	458 (64.33%)		
Yes	238 (23.92%)	70 (26.52%)	103 (26.41%)	254 (35.67%)		
**Cancer**					0.598	-
No	967 (97.19%)	255 (96.59%)	383 (98.21%)	691 (97.05%)		
Yes	28 (2.81%)	9 (3.41%)	7 (1.79%)	21 (2.95%)		
**Associated injuries**					0.417	-
No	931 (93.57%)	244 (92.42%)	368 (94.36%)	655 (91.99%)		
Yes	64 (6.43%)	20 (7.58%)	22 (5.64%)	57 (8.01%)		
**Dementia**					<0.001	-
No	982 (98.69%)	248 (93.94%)	377 (96.67%)	665 (93.40%)		
Yes	13 (1.31%)	16 (6.06%)	13 (3.33%)	47 (6.60%)		
**COPD**					0.201	-
No	942 (94.67%)	254 (96.21%)	361 (92.56%)	667 (93.68%)		
Yes	53 (5.33%)	10 (3.79%)	29 (7.44%)	45 (6.32%)		
**Hepatitis**					0.775	-
No	969 (97.39%)	257 (97.35%)	377 (96.67%)	688 (96.63%)		
Yes	26 (2.61%)	7 (2.65%)	13 (3.33%)	24 (3.37%)		
**Gastritis**					0.352	-
No	973 (97.79%)	258 (97.73%)	386 (98.97%)	702 (98.60%)		
Yes	22 (2.21%)	6 (2.27%)	4 (1.03%)	10 (1.40%)		
**Treatment strategy**					<0.001	-
CRIF/ORIF	784 (78.79%)	199 (75.38%)	278 (71.28%)	463 (65.03%)		
HA	196 (19.70%)	60 (22.73%)	104 (26.67%)	242 (33.99%)		
THA	15 (1.51%)	5 (1.89%)	8 (2.05%)	7 (0.98%)		
**Time to operation (d)**	4.24 ± 2.32	4.62 ± 2.49	4.38 ± 2.08	4.18 ± 3.10	0.094	<0.001
**Operation time (m)**	94.08 ± 35.93	93.28 ± 39.33	92.82 ± 35.30	91.70 ± 35.04	0.606	0.448
**Blood loss (mL)**	200 (50–1600)	200 (50–1500)	200 (50–1000)	200 (20–1200)	0.424	0.867
**Stay in hospital (d)**	8.52 ± 3.33	8.78 ± 2.99	8.49 ± 2.86	8.99 ± 3.81	0.021	0.044
**Follow up (m)**	41.94 ± 18.65	40.96 ± 19.57	38.40 ± 19.03	37.44 ± 19.04	<0.001	<0.001
**2-Year Mortality**					<0.001	-
Survival	867 (87.14%)	222 (84.09%)	313 (80.26%)	564 (79.21%)		
Deceased	128 (12.86%)	42 (15.91%)	77 (19.74%)	148 (20.79%)		
**Long-term Mortality**					<0.001	-
Survival	741 (74.47%)	179 (67.80%)	263 (67.44%)	445 (62.50%)		
Deceased	254 (25.53%)	85 (32.20%)	127 (32.56%)	267 (37.50%)		

^†^ *p*-value for parameter test. * *p*-value for non-parameter test. For continuous variables, we used the Kruskal–Wallis rank-sum test; for count variables with a theoretical count < 10, we used Fisher’s exact test. **Abbreviation: TTA**, time to admission**; CHD**, coronary heart disease; **COPD**, chronic obstructive pulmonary disease; **HA**, hemiarthroplasty; **CRIF/ORIF**, closed/open reduction and internal fixation; **THA**, total hip arthroplasty.

**Table 2:** The multivariate Cox regression analysis between TTA and long-term mortality.

	Non-Adjusted	Adjust I	Adjust II
**TTA (h)**	1.000 (1.000, 1.001) 0.009	1.000 (1.000, 1.000) 0.0147	1.000 (1.000, 1.000) 0.040
**TTA (h) categorical**			
TTA ≤ 6 h	1	1	1
6 h < TTA ≤ 12 h	1.292 (1.011, 1.652) 0.041	1.298 (1.015, 1.660) 0.037	1.223 (0.955, 1.568) 0.111
12 h < TTA ≤ 24 h	1.392 (1.125, 1.723) 0.002	1.266 (1.022, 1.567) 0.031	1.250 (1.008, 1.550) 0.042
TTA > 24 h	1.649 (1.389, 1.958) <0.001	1.575 (1.326, 1.871) <0.001	1.489 (1.249, 1.776) <0.001
***p*** **for trend**	<0.001	<0.001	<0.001

**Data in the table:** HR (95% CI) **Outcome variate**: Mortality **Exposure variates**: TTA; TTA categorical; TTA categorical continuous **Adjust model I** adjusted for age, sex **Adjust model II** adjusted for age, sex, CHD, arrhythmia, ischemic stroke, cancer, dementia, COPD, hepatitis, and time to operation, stay in hospital, fracture classification, and treatment strategy.

**Table 3:** The nonlinear association between TTA and long-term mortality.

Outcome:	Mortality
**Model**	
**Cox proportional hazards regression model**	1.000 (1.000, 1.000) 0.040
**The two-piecewise Cox proportional hazards regression model**	
Inflection point (K)	24
<K	1.016 (1.008, 1.024) < 0.001
>K	1.000 (1.000, 1.000) 0.531
***p*** **-value for log-likelihood ratio test**	<0.001
95% CI of the Inflection point	21, 28

**Data in the table:** HR (95% CI) **Outcome variate**: Mortality **Exposure variates**: TTA **Adjust variables**: age, sex, CHD, arrhythmia; ischemic stroke, cancer, dementia, COPD, hepatitis, and time to operation, stay in hospital, fracture classification, and treatment strategy.

**Table 4:** Stratification analysis result of subgroups.

Subgroups	No. of Patients	Inflection Point (h)	HR (95% CI) *p*-Value < Inflection Point	HR (95% CI) *p*-Value > Inflection Point	*p* for Log-Likelihood Ratio Test
**Age (y)**					
65 ≤ age < 80	1130	312	1.003 (1.001, 1.004) < 0.001	0.999 (0.998, 1.001) 0.369	<0.001
age ≥ 80	1229	48	1.008 (1.003, 1.013) 0.002	1.000 (0.999, 1.001) 0.945	0.002
**Fracture classification**					
Intertrochanteric fracture	1745	11	1.037 (1.013, 1.062) 0.003	1.000 (1.000, 1.001) 0.067	0.003
Femoral neck fracture	616	48	1.017 (1.008, 1.027) < 0.001	0.999 (0.999, 1.000) 0.243	<0.001
**CHD**					
Yes	1215	11	1.048 (1.018, 1.079) 0.002	1.000 (1.000, 1.001) 0.097	0.001
No	1144	36	1.012 (1.004, 1.021) 0.005	1.000 (0.999, 1.000) 0.296	0.004
**Arrhythmia**					
Yes	757	12	1.039 (1.007, 1.073) 0.018	1.000 (0.999, 1.001) 0.999	0.017
No	1602	72	1.007 (1.004, 1.011) < 0.001	1.000 (0.999, 1.001) 0.713	<0.001
**Ischemic stroke**					
Yes	665	48	1.007 (1.000, 1.014) 0.054	1.001 (1.000, 1.001) 0.022	0.085
No	1694	264	1.002 (1.001, 1.003) < 0.001	0.999 (0.999, 1.000) 0.130	<0.001
**Cancer**					
Yes	65	12	1.138 (1.019, 1.271) 0.021	0.999 (0.997, 1.001) 0.157	0.015
No	2294	72	1.005 (1.002, 1.008) 0.002	1.000 (0.999, 1.000) 0.638	<0.001
**Dementia**					
Yes	89	4	1.971 (0.670, 5.799) 0.218	1.002 (1.000, 1.004) 0.032	0.139
No	2270	17	1.026 (1.013, 1.038) < 0.001	1.000 (0.999, 1.000) 0.868	<0.001
**COPD**					
Yes	137	2	0.793 (0.104, 6.069) 0.824	1.000 (0.998, 1.002) 0.750	0.829
No	2222	72	1.006 (1.003, 1.009) < 0.001	1.000 (0.999, 1.000) 0.920	<0.001
**Hepatitis**					
Yes	70	6	1.406 (1.056, 1.871) 0.020	0.999 (0.997, 1.001) 0.503	0.011
No	2289	72	1.006 (1.003, 1.009) < 0.001	1.000 (0.999, 1.000) 0.936	<0.001
**Time to operation (d)**					
<3	556	312	1.002 (1.001, 1.004) 0.003	1.000 (0.999, 1.001) 0.793	0.009
≥3	1803	20	1.021 (1.001, 1.032) < 0.001	1.000 (0.999, 1.000) 0.624	<0.001

## Data Availability

Xi’an Honghui Hospital implemented the data. According to relevant regulations, the data cannot be shared but can be requested from the corresponding author.

## Supplementary Materials

The following supporting information can be downloaded at: https://www.mdpi.com/article/10.3390/jcm15020752/s1; Table S1: The general information and baseline characteristics of patients lost to follow-up and follow-up; Table S2: Univariate analysis of the association between variables and long-term mortality, and the VIF for all variables; Table S3: The nonlinearity association between TTA and 2-year mortality.

## Abbreviations

TTA, time to admission; CHD, coronary heart disease; COPD, chronic obstructive pulmonary disease; HA, hemiarthroplasty; CRIF/ORIF, closed/open reduction and internal fixation; THA, total hip arthroplasty.
