# Supplementary material for: Threshold Effect of Time to Admission on Long-Term Mortality in Geriatric Hip Fractures: A 24-H Critical Window Identified
Source: J Clin Med. 2026 Jan 16;15(2):752. doi: 10.3390/jcm15020752 (PMC12842464; doi:10.3390/jcm15020752)
Supplement: Supplementary file 1 [file jcm-15-00752-s001.zip › jcm-4087537-supplementary.pdf]

Supplementary Table S1. The general information and baseline characteristics of patients lost to follow-up and follow-up.

Groups	Lost to follow-up	Follow-up	Standardize diff.	*P*-value
**N**	478	2361		
**Age (year)**	79.90±6.56	79.44±6.71	0.07 (-0.03, 0.17)	0.176
**Sex**			0.01 (-0.09, 0.11)	0.888
Male	152 (31.80%)	743 (31.47%)		
Female	326 (68.20%)	1618 (68.53%)		
**Injury mechanism**			0.07 (-0.03, 0.17)	0.467
Falling	467 (97.70%)	2286 (96.82%)		
Accident	10 (2.09%)	61 (2.58%)		
Other	1 (0.21%)	14 (0.59%)		
**Fracture classification**			0.02 (-0.08, 0.12)	0.654
Intertrochanteric fracture	358 (74.90%)	1745 (73.91%)		
Femoral neck fracture	120 (25.10%)	616 (26.09%)		
**Hypertension**			0.01 (-0.09, 0.11)	0.844
No	242 (50.63%)	1207 (51.12%)		
Yes	236 (49.37%)	1154 (48.88%)		
**Diabetes**			0.05 (-0.05, 0.15)	0.344
No	394 (82.43%)	1902 (80.56%)		
Yes	84 (17.57%)	459 (19.44%)		
**CHD**			0.00 (-0.10, 0.10)	0.999
No	232 (48.54%)	1146 (48.54%)		
Yes	246 (51.46%)	1215 (51.46%)		
**Arrhythmia**			0.07 (-0.02, 0.17)	0.136
No	308 (64.44%)	1604 (67.94%)		
Yes	170 (35.56%)	757 (32.06%)		
**Hemorrhagic stroke**			0.03 (-0.07, 0.12)	0.606
No	470 (98.33%)	2313 (97.97%)		
Yes	8 (1.67%)	48 (2.03%)		
**Ischemic stroke**			0.00 (-0.10, 0.10)	0.953
No	344 (71.97%)	1696 (71.83%)		
Yes	134 (28.03%)	665 (28.17%)		
**Cancer**			0.05 (-0.05, 0.14)	0.339
No	461 (96.44%)	2296 (97.25%)		
Yes	17 (3.56%)	65 (2.75%)		
**Associated injuries**			0.02 (-0.07, 0.12)	0.624
No	442 (92.47%)	2198 (93.10%)		
Yes	36 (7.53%)	163 (6.90%)		
**Dementia**			0.09 (-0.01, 0.18)	0.112
No	467 (97.70%)	2272 (96.23%)		
Yes	11 (2.30%)	89 (3.77%)		
**COPD**			0.03 (-0.07, 0.13)	0.564
No	447 (93.51%)	2224 (94.20%)		
Yes	31 (6.49%)	137 (5.80%)		
**Hepatitis**			0.03 (-0.06, 0.13)	0.494
No	461 (96.44%)	2291 (97.04%)		
Yes	17 (3.56%)	70 (2.96%)		
**Gastritis**			0.02 (-0.07, 0.12)	0.63
No	471 (98.54%)	2319 (98.22%)		
Yes	7 (1.46%)	42 (1.78%)		
**Treatment strategy**			0.05 (-0.05, 0.15)	0.63
ORIF	355 (74.27%)	1724 (73.02%)		
HA	114 (23.85%)	602 (25.50%)		
THA	9 (1.88%)	35 (1.48%)		
**Time to operation (d)**	4.24±2.48	4.29±2.57	0.02 (-0.08, 0.12)	0.729
**Time to admission (h)**	95.81±233.02	79.15±237.19	0.07 (-0.03, 0.17)	0.16
**Operation time (mins)**	92.57±34.46	93.07±35.95	0.01 (-0.08, 0.11)	0.782
**Blood loss (mL)**	233.82±149.78	241.32±153.49	0.05 (-0.05, 0.15)	0.337
**Infusion (mL)**	1555.00±403.56	1556.04±383.40	0.00 (-0.10, 0.10)	0.958

Stay in hospital (d)	9.06±3.70	8.69±3.38	0.11 (0.01, 0.20)	0.03

Supplementary Table S2. Univariate analysis of the association between variables and long-term mortality, and the VIF for all variables.

	Statistics	HR (95%CI) *P*-value	VIF step 1	VIF step 2
**Age (year)**	79.44 ± 6.71	1.08 (1.06, 1.09) <0.0001	1.1	1.1
**Sex**			1.1	1.1
Male	743 (31.47%)	1		
Female	1618 (68.53%)	0.74 (0.63, 0.86) <0.0001		
**Injury mechanism**			1	1
Falling	2286 (96.82%)	1		
Accident	61 (2.58%)	0.30 (0.14, 0.64) 0.0018		
Other	14 (0.59%)	1.49 (0.67, 3.32) 0.3325		
**Fracture classification**			1.2	1.2
Intertrochanteric fracture	1745 (73.91%)	1		
Femoral neck fracture	616 (26.09%)	0.98 (0.82, 1.18) 0.8500		
**Hypertension**			1.1	1.1
No	1207 (51.12%)	1		
Yes	1154 (48.88%)	1.11 (0.96, 1.28) 0.1596		
**Diabetes**			1.1	1.1
No	1902 (80.56%)	1		
Yes	459 (19.44%)	1.02 (0.84, 1.22) 0.8672		
**CHD**			1.1	1.1
No	1146 (48.54%)	1		
Yes	1215 (51.46%)	1.20 (1.04, 1.39) 0.0136		
**Arrhythmia**			1.1	1.1
No	1604 (67.94%)	1		
Yes	757 (32.06%)	1.21 (1.04, 1.40) 0.0150		
**Hemorrhagic stroke**			1	1
No	2313 (97.97%)	1		
Yes	48 (2.03%)	0.95 (0.56, 1.61) 0.8426		
**Ischemic stroke**			1.1	1.1
No	1696 (71.83%)	1		
Yes	665 (28.17%)	1.36 (1.16, 1.59) 0.0001		
**Cancer**			1	1
No	2296 (97.25%)	1		
Yes	65 (2.75%)	1.66 (1.15, 2.39) 0.0067		
**Associated injuries**			5.6	NA†
No	2198 (93.10%)	1		
Yes	163 (6.90%)	0.86 (0.64, 1.17) 0.3457		
**Dementia**			1	1
No	2272 (96.23%)	1		
Yes	89 (3.77%)	2.54 (1.91, 3.37) <0.0001		
**COPD**			1	1
No	2224 (94.20%)	1		
Yes	137 (5.80%)	1.47 (1.12, 1.93) 0.0057		
**Hepatitis**			1	1
No	2291 (97.04%)	1		
Yes	70 (2.96%)	1.48 (1.03, 2.14) 0.0346		
**Gastritis**			1	1
No	2319 (98.22%)	1		
Yes	42 (1.78%)	0.83 (0.47, 1.46) 0.5102		
**Stay in hospital (d)**	8.69 ± 3.38	1.02 (1.00, 1.04) 0.0764	1.5	1.5
**Time to admission (h)**	79.15 ± 237.19	1.00 (1.00, 1.00) 0.0094	1.1	1.1

**Time to operation (d)**	4.29 ± 2.57	1.02 (0.99, 1.05) 0.1420	1.5	1.5
**Treatment strategy**			5.6	1
ORIF	1724 (73.02%)	1		
HA	602 (25.50%)	1.08 (0.91, 1.29) 0.3879		
THA	35 (1.48%)	0.20 (0.05, 0.82) 0.0249		
**Operation time (mins)**	93.07 ± 35.95	1.00 (1.00, 1.00) 0.1577	1.2	1.2
**Blood loss (mL)**	241.32 ± 153.49	1.00 (1.00, 1.00) 0.5615	1.2	1.2

†The excluded variable according to the criterion of VIF < 4.

VIF stepwise screening method: First, calculate the VIF for each variable. If the maximum VIF value is ⩾ 4, remove the variable with the largest VIF. Next, repeat the previous step until the VIF values of all remaining variables are less than 4.

Supplementary Table S3. The nonlinearity association between TTA and 2-year mortality.

**Outcome:**	**Mortality**
**Model**	
**Logistical regression model**	1.000 (1.000, 1.001) 0.080
**The two-piecewise logistical regression model**	
Inflection point (K)	17
< K	1.032 (1.013, 1.051) 0.001
> K	1.000 (1.000, 1.001) 0.487
***P* value for log-likelihood ratio test**	<0.001
**95%CI of Inflection point**	9,22

Data in the table: OR (95% CI) **Outcome variate**: 2 year mortality **Exposure variates**: TTA **Adjust variables**: age, sex, CHD, arrhythmia; ischemic stroke, cancer, dementia, COPD, hepatitis, and time to operation, stay in hospital, fracture classification and treatment strategy.
